# Nucleosome positioning based on DNA sequence embedding and deep learning

**DOI:** 10.1186/s12864-022-08508-6

**Published:** 2022-04-13

**Authors:** Guo-Sheng Han, Qi Li, Ying Li

**Affiliations:** 1 Department of Mathematics and Computational Science, Xiangtan University, Xiangtan, 411105 Hunan China; 2 Key Laboratory of Intelligent Computing and Information Processing of Ministry of Education and Hunan Key Laboratory for Computation and Simulation in Science and Engineering, Xiangtan University, Xiangtan, 411105 Hunan China; 3 Xiangtan Medicine Health Vocational College, Xiangtan, 411102 Hunan China

**Keywords:** Nucleosome positioning, Word vector, Deep learning, Convolutional neural network, Bidirectional recurrent neural network

## Abstract

**Background:**

Nucleosome positioning is the precise determination of the location of nucleosomes on DNA sequence. With the continuous advancement of biotechnology and computer technology, biological data is showing explosive growth. It is of practical significance to develop an efficient nucleosome positioning algorithm. Indeed, convolutional neural networks (CNN) can capture local features in DNA sequences, but ignore the order of bases. While the bidirectional recurrent neural network can make up for CNN's shortcomings in this regard and extract the long-term dependent features of DNA sequence.

**Results:**

In this work, we use word vectors to represent DNA sequences and propose three new deep learning models for nucleosome positioning, and the integrative model NP_CBiR reaches a better prediction performance. The overall accuracies of NP_CBiR on H. sapiens, C. elegans, and D. melanogaster datasets are 86.18%, 89.39%, and 85.55% respectively.

**Conclusions:**

Benefited by different network structures, NP_CBiR can effectively extract local features and bases order features of DNA sequences, thus can be considered as a complementary tool for nucleosome positioning.

## Background

In eukaryotes, nucleosomes are the basic structural unit of chromatin. The nucleosome is composed of a histone octamer core which is formed by four types of histones (H2A, H2B, H3, H4) and DNA that is tightly wound around histone core about 1.65 turns. The winding DNA is called core DNA with 147 bp in length. The DNA that binds to histone H1 and connects two adjacent nucleosomes is called linker DNA, in around 20–60 bp, and it is responsible for stabilizing the structure of nucleosomes [1]. Nucleosomes not only compress the chromatin structure, but also play a key role in biological processes such as genome expression, DNA replication and repair [2–5]. Therefore, it is of far-reaching biological significance to study nucleosome positioning on the whole genome.

Since DNA needs to be bent and coiled around histone core, the flexible regions of DNA are more likely to form nucleosomes [6]. In the core DNA region found in chicken red blood cells, AA / TT / TA fragments repeat every 10 bp in the direction of the DNA facing to histone core; GG/GC/CC/CG appears every 10 bp in the direction of the back of histone core [7]. Similar periodic laws have been found in the studies of other eukaryotes [8]. In addition, the study found that nucleosomes in the poly (dA:dT) region were significantly lacking [9]. The affinity between DNA and histones obviously depends on the order of the bases, which indicates that DNA sequences do affect the formation of nucleosomes [10]. Peckham et al. extract the k-mer frequency of the DNA sequence and use a support vector machine to clearly distinguish the core DNA and junction DNA sequences of the yeast [11]. These researches indicate to a certain extent that nucleosome positioning is affected by sequence information. Thence, we can construct theoretical models to extract sequence features and distinguish core DNA from linker DNA to predict the location of nucleosomes.

In the past decade, due to the popularity of machine learning, more nucleosome positioning prediction models based on DNA sequence information have been proposed [12–17]. In addition, with the widespread popularity of artificial intelligence, deep learning algorithms have also been applied to nucleosome positioning and made great progress. Di Gangi et al. utilize a stacked convolutional layer and long-short-term memory (LSTM) network to establish a deep learning model [18]. LeNup add the Inception module and gated convolutional structure to the convolutional neural network (CNN) [19]. CORENup conduct the parallel method of CNN and LSTM network to show high performance in both classification accuracy and calculation time [20]. These deep learning prediction models all use one-hot encoding to represent DNA sequences.

DNA sequence is composed of A, T, C, and G, and can be seen as a broad language which natural language processing (NLP) technology can be applied to. Word2vec is a technology that converts a single word into a vector, which is mainly used in the field of NLP [21]. It also has a good application on biological sequence processing. Ng utilize the human genome sequence as the learning corpus to exploit the pre-training vector of the DNA sequence (dna2vec) through training word2vec model [22]. Dna2vec has been used to predict the interaction between enhancer and promoter [23]. In predicting the compound-protein interaction, the word2vec method was also used to obtain the word vector of the amino acid sequence [24].

CNN has obvious advantages in image processing. It was initially mainly used in the field of computer vision. In 2014, TextCNN model used convolutional neural networks in text classification tasks, and selected multiple filters of different scales to extract more local information of the text, and the effectiveness was verified [25]. The sequence of bases contains rich information, and there are long-range interactions between each base. Therefore, recurrent neural network (RNN) could be helpful to mine the hidden information in the DNA sequence [26]. Gated recurrent unit (GRU) and long short-term memory (LSTM) networks are two mainstream variants of RNN, which can learn information from a long time ago [18, 23].

In this paper, we utilized the k-mer embedding trained by word2vec to represent the DNA sequence. In addition, we built several deep learning models to compare the impact of different network structures on prediction quality. We found that the prediction performance of the hybrid model that integrates CNN and RNN is significantly better than single structure model. Our results also demonstrated that using the k-mer vector to represent the DNA sequence is more effective.

## Results and discussion

### Selection of word vector dimensions

Obviously, the size of k-mer will determine the vocabulary size, then affect the training efficiency. In addition, we also need to notice the dimension of word vector especially. The setting of vector dimension is related to the vocabulary size and experimental requirements. The higher dimensional word vector can more accurately reflect the feature distribution of each k-mer in the sequence space. However, the higher word vector dimension is, the more calculation burden becomes.

In order to determine k and word vector dimension, we train k-mers into word vectors with several different dimensions, for k ranging from 3 to 6 respectively. Then, word vectors of different dimensions are fed to support vector machine (SVM) to find the most suitable k and word vector dimension.

**Fig. 1:**
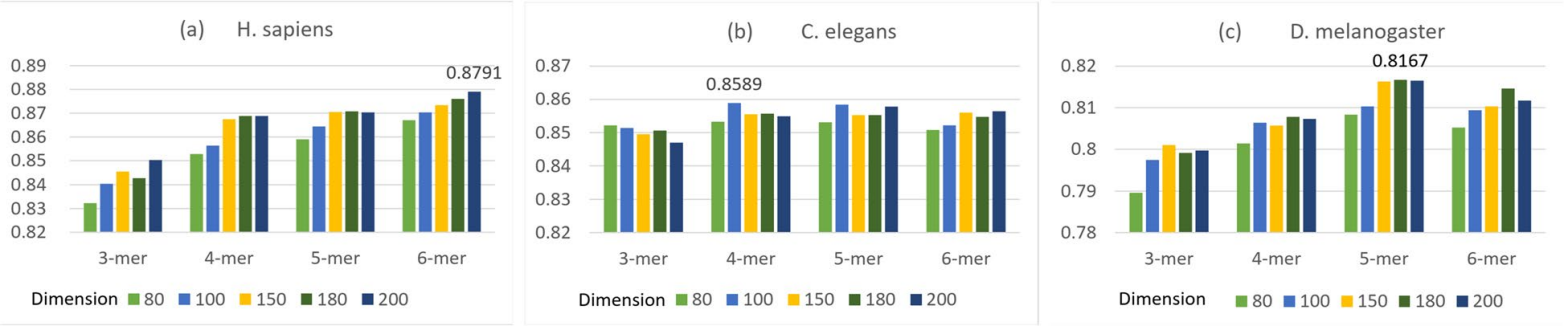
The histograms show the overall accuracy of nucleosome positioning by using SVM with different k and vector dimensions. **a** H. sapiens achieves the highest classification accuracy with k = 6 and vector dimension of 200; **b** C. elegans achieves the highest classification accuracy, with k = 4 and vector dimension of 100; **c** D. melanogaster achieves the highest classification accuracy, with k = 5 and vector dimension of 180

In this paper, we applied python package (gensim 3.8.3) to implement the word2vec model. And we used python package Scikit-learn (Sklearn 0.23) to implement the SVM algorithm. Figure 1 shows the experimental results with combinations of different k and dimensions on the first group of datasets.

In summary, the selection of k and vector dimensions for each species in this experiment are shown in Table 1.

**Table 1:** DNA sequence vector dimension setting

Species	k-mer	Vector dimension
H. sapiens	6	200
C. elegans	4	100
D. melanogaster	5	180

### CNN model improves the classification performance

We compare the classification results of CNN model with SVM, as shown in Table 2. For each species, the bold numbers in the table indicate the better model under each evaluation index.

**Table 2:** Classification results of SVM and CNN via tenfold cross validation

Species		H. sapiens	C. elegans	D. melanogaster
SVM	ACC	**0.8791**	0.8589	0.8167
	Sn	**0.9059**	0.8944	0.7928
	Sp	**0.8526**	0.824	0.8411
	MCC	**0.7601**	0.7202	0.6346
CNN	ACC	0.8443	**0.8812**	**0.8247**
	Sn	0.879	**0.9151**	**0.8231**
	Sp	0.81	**0.8478**	**0.8263**
	MCC	0.6958	**0.7664**	**0.6546**

Table 2 shows that prediction performance of CNN on C. elegans and D. melanogaster are significantly better than SVM. Especially for C. elegans dataset, CNN is higher than SVM in ACC, ${S}_{n}$, ${S}_{p}$, MCC by 2.23%, 2.07%, 2.38%, 4.62%, respectively. However, for H. sapiens dataset, CNN is lower than SVM in ACC, ${S}_{n}$, ${S}_{p}$, MCC by 3.48%, 2.69%, 4.26%, 6.43%.

### Performance on BiGRU + BiLSTM model is close to CNN

The performance of the BiGRU + BiLSTM model is also evaluated by tenfold cross-validation, which is shown in Table 3.

**Table 3:** The prediction quality of BiGRU + BiLSTM via tenfold cross validation

Species	ACC	Sn	Sp	MCC
H. sapiens	0.8428	0.8891	0.797	0.6917
C. elegans	0.8817	0.9119	0.8520	0.7666
D. melanogaster	0.8285	0.7714	0.8867	0.6629

Compared with Table 2, we find that results obtained by these two deep learning models are relatively close, and the difference in accuracy is less than 0.4%. Overall, SVM has obvious advantages for H. sapiens datasets.

### The integrative model NP_CBiR yields outstanding performance

NP_CBiR is based on convolutional layers, BiGRU and BiLSTM networks. Table 4 shows classification results of NP_CBiR via tenfold cross-validation.

**Table 4:** The prediction performance of NP_CBiR via tenfold cross validation

Species	ACC	Sn	Sp	MCC	AUC
H. sapiens	0.8618	0.8909	0.8330	0.7284	0.9234
C. elegans	0.8939	0.9427	0.8459	0.7924	0.9530
D. melanogaster	0.8555	0.8769	0.8337	0.7119	0.9251

NP_CBiR has improved prediction performance on each dataset compared with the previous two deep learning model in Tables 2 and 3. Except H. sapiens on which the classification results of NP_CBiR are little lower than SVM, the performance of NP_CBiR on the other two species are all higher than SVM. More precisely, the ACC of NP_CBiR for H. sapiens, C. elegans, and D. melanogaster datasets are 1.9%, 1.2%, and 2.7% higher than the BiGRU + BiLSTM model, respectively. These results show that the performance of hybrid model is better.

**Fig. 2:**
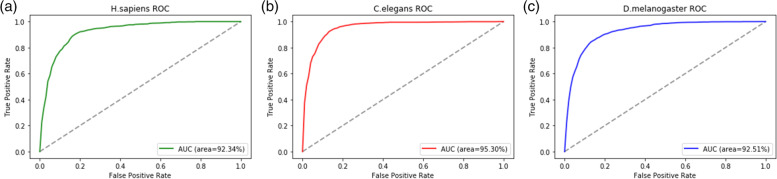
The ROC curves show the performance of NP_CBiR. **a** AUC is 0.9234 for H. sapiens; **b** AUC is 0.953 for C. elegans; **c** AUC is 0.9251 for D. melanogaster

We also plot the ROC curves of NP_CBiR on the first set of data, as shown in Fig. 2.

### Comparison with other algorithms

The above results show that the prediction performance of jointly using convolutional layers and RNN networks is significantly better than single module neural network. Therefore, we further compare NP_CBiR with other proposed nucleosome positioning algorithms on the second group of datasets. Liu et al. [27] proposed an evaluation method for this group of datasets. The method stipulates that 100 test sample sets are randomly selected from each dataset, and each sample set contains 100 core DNA sequences and 100 linker DNA sequences, then calculates the ROC curve of each sample set and the average of the 100 sample sets.

The experimental results are shown in Table 5, the approximate value is represented by "∼", and the bold number represents the best value. The second column of the table shows the best AUC values of the eight methods reported by Liu et al. [27].

**Table 5:** Experimental results of the second dataset

Dataset	Best for Liu	DLNN	CORENup	NP_CBiR
H-5U	∼0.7	0.68	0.760	**0.78**
H-LC	∼0.65	0.81	0.910	**0.92**
H-PM	0.67	0.77	**0.875**	0.86
D-5U	∼0.7	0.67	**0.746**	0.71
D-LC	∼0.7	0.71	**0.736**	0.72
D-PM	∼0.7	0.73	0.738	**0.74**

Due to the limitation of table size, the species name is indicated by an abbreviation. *H H* Sapiens, *D D* Melanogaster, *LC* Largest chromosome, *5U* 5’UTR exon region, *PM*  Promoter

For NP_CBiR, its AUC value on the H-5U, H-LC, and D-PM are better than other methods; the AUC value on the H-PM, D-5U, and D-LC are better than the results of Liu et al. [27] and DLNN [18], but slightly lower than CORENup [20].

We compare the classification results of NP_CBiR model with SVM, as shown in Table 6. For each Dataset, the bold numbers in the table indicate the better model.

**Table 6:** Classification results of SVM and NP_CBiR

Dataset	H-5U	H-LC	H-PM	D-5U	D-LC	D-PM
SVM	**0.8123**	**0.9216**	**0.8762**	0.6890	0.7128	0.7294
NP_CBiR	0.78	**0.92**	0.86	**0.71**	**0.72**	**0.74**

**Table 7:** Comparison of NP_CBiR with other methods on H. sapiens

Method	ACC	Sn	Sp	MCC	AUC
DLNN	0.8537	0.8834	0.8229	-	-
ZCMM	0.7772	0.7487	0.8151	0.5600	0.8610
NP_CBiR	**0.8618**	**0.8909**	**0.8330**	**0.7284**	**0.9234**

**Table 8:** Comparison of NP_CBiR with other methods on C. elegans

Method	ACC	Sn	Sp	MCC	AUC
DLNN	**0.8962**	0.9304	**0.8634**	-	-
ZCMM	0.8534	0.7880	0.8410	0.6200	0.9120
NP_CBiR	0.8939	**0.9427**	0.8459	**0.7924**	**0.9530**

**Table 9:** Comparison of NP_CBiR with other methods on D. melanogaster

Method	ACC	Sn	Sp	MCC	AUC
DLNN	0.8560	0.8781	0.8333	-	-
ZCMM	**0.9362**	**0.9226**	0.7964	0.7000	0.9110
NP_CBiR	0.8555	0.8769	**0.8337**	**0.7119**	**0.9251**

Table 6 shows that prediction performance of NP_CBiR on D-5U, D-LC and D-PM are slightly better than SVM, on H-LC is flat with SVM, and on H-5U and H-PM are slightly lower than SVM.

In addition, we compared the prediction results of NP_CBiR with other methods in the first group dataset via tenfold cross-validation. As shown in Table 7, 8 and 9, the best values are in bold.

Compared with other algorithms, for H. sapiens, the classification accuracy of NP_CBiR is higher than DLNN and ZCMM by 0.81% and 8.46%. For C. elegans, the prediction result of the NP_CBiR is close to DLNN, and it is higher than ZCMM in ACC,${S}_{n}$, ${S}_{p}$, MCC, AUC by 4.05%, 15.47%, 0.49%, 17.24%, 4.10%, respectively. For D. melanogaster, the ZCMM still performed best, and the prediction quality of NP_CBiR is comparable to DLNN.

We trained our model NP_CBiR using H. sapiens.LC of Table 11 as the training set. Then the trained model makes predictions under the real context of the whole genome (hg38) reference to Healthy_Song data. The overall classification accuracy of NP_CBiR is 65.12%.

These results show that the combination of CNN, BiGRU and BiLSTM network can make up for the shortcomings of a single module network model and effectively improves the classification performance.

## Conclusions

In this work, nucleosome positioning method based on DNA sequence embedding and deep learning is introduced. Word vector embedding of DNA sequence has been verified to be helpful in nucleosome positioning. Moreover, we construct three deep learning models with different network structures to better understand advantages of these structures. Our results demonstrate that NP_CBiR model which integrated convolutional layers, BiGRU and BiLSTM network structures has a better prediction performance. Convolutional layers can extract local features in DNA sequences, but ignore the order of bases and lose the hidden position information. While BiGRU and BiLSTM networks can make up for CNN’s shortcomings in this regard, they take the contextual information into account and thus can dig out the correlation information in the sequence. The prediction performance of NP_CBiR to a certain degree is comparable with or better than SVM. Therefore, by combining these two structures, the hybrid model NP_CBiR can effectively extract the local features and long-term dependent features of the sequence and be considered as a complementary model in distinguishing core DNA from linker DNA.

Nucleosome positioning is a complex dynamic process, it still needs to be further researched. In recent years, many excellent and effective models have emerged with the continuous development of deep learning. The proposed models in this paper contain relatively simple architectures. As for future work, we will explore the application of more advanced neural networks and models in nucleosome positioning.

## Methods

In this work, we segment a DNA sequence to several k-mers [15], and then apply word2vec model to transform k-length sub-sequence of DNA sequence into the word vectors. Meanwhile, we utilize support vector machine (SVM) to determine the best dimension of the DNA word vector. Then we propose three nucleosome positioning deep learning models with different networks, such as CNN, BiGRU and BiLSTM. In addition, we conduct relatively sufficient experiments for each model to compare and analyze the prediction performance among models. We choose PaddlePaddle deep learning framework to implement related experiments (https://www.paddlepaddle.org.cn).

### Dataset descriptions

This paper mainly uses two groups of datasets downloaded from published papers. The first datasets contain DNA sequence data of H. sapiens, C. elegans, D. melanogaster and D. melanogaster, they were constructed by Guo et al. [12], the length of sequences is 147 bp.) The yeast data was constructed by Chen et al. [28], which is 150 bp in length. In order to avoid redundancy and reduce homology deviation, sequences with more than 80% similarity were eliminated. The core DNA sequences are positive samples (P-S), and linker DNA sequences are negative samples (N-S). The sample size of the first dataset sequence is shown in the Table 10.

**Table 10:** Statistical information of the first datasets

Species	P-S	N-S	Total
H. sapiens	2273	2300	4573
C. elegans	2567	2608	5175
D. melanogaster	2900	2850	5750

The second datasets are from Liu et al. [27]. It contains six subsets of DNA sequences related to two species. They are largest chromosome (LC), promoter (PM) and 5’UTR exon region (5U) sequences from H. sapiens and D. melanogaster. Based on the experimental data provided by Liu, Amato et al. [20] extracted core DNA and linker DNA by downloading the genome file from the UCSC gene browse http://www.genome.ucsc.edu/cgi-bin/hgTables. The length of sequences is 147 bp and sample sizes of the second group of datasets are shown in the Table 11.

**Table 11:** Statistical information of the second dataset

Species	region	P-S	N-S	Total
H. sapiens	LC	97,209	65,563	162,772
	PM	56,404	44,639	101,043
	5U	11,769	4880	16,649
D. melanogaster	LC	46,054	30,458	76,512
	PM	48,251	28,763	77,014
	5U	4669	2704	7373

In addition, we downloaded an additional set of Homo sapiens genome sequences containing nucleosome references to implement the genome-wide test to obtain the predictive performance of our model under the real context. We downloaded Healthy_Song data (GSE81314_healthy_Song_stable_100bp_hg38.bed.gz) from GRCh38(hg38) via https://generegulation.org/NGS/stable_nucs/hg38/, and expanded the length of sequence from 100 to 147 bp. The number of nucleosome sequences is 404565.

### Performance evaluation

In this work, we adopted k-fold cross validation (for k = 10) to train and assess the model. Original dataset is divided into k mutually disjunct parts, k-1 parts for training and 1 part for testing. The train/assess-procedure will be conducted k times for k different testing parts, and the average performance on these k testing parts can be seen as model’s generalization ability. In classification tasks, it is necessary to set metrics to evaluate the generalization ability of the model. Usually, we use sensitivity (${S}_{n}$), specificity (${S}_{p}$), accuracy (ACC), and Matthew’s correlation coefficient (MCC) to measure the effectiveness of the model [12, 19]. The mathematical expressions are:

1 $$\left\{\begin{array}{c}{S}_{n}=\frac{TP}{TP+FN}\\ {S}_{p}=\frac{TN}{TN+FP}\\ ACC=\frac{TP+TN}{TP+TN+FP+FN}\\ MCC=\frac{TP\times TN-FP\times FN}{\sqrt{(TP+FN)\times (TP+FP)\times (TN+FN)\times (TN+FP)}}\end{array}\right.$$

### DNA sequences embedding based on word2vec

One-hot encoding is often used in deep learning to represent DNA sequences [18–20]. This method has a limitation that vectors are independent each other so that the model cannot capture the hidden association information in the sequence. While word2vec model that trained by context information maps each word into a dense continuous low-dimensional word vector [22, 29], which can generate word vector reflecting the connection between words. Word2vec makes up for the defect that one-hot encoding cannot express the similarity between words. Meanwhile, it has the advantages of simple model hierarchy and short training time. Word2vec's basic structure is a shallow neural network with two types of training modes: Continuous Bag-of-Words (CBOW) and Skip-gram. In practice, Skip-gram has a better processing effect on low-frequency words. Therefore, we choose Skip-gram model to train the DNA sequence word vector in this paper.

**Fig. 3:**
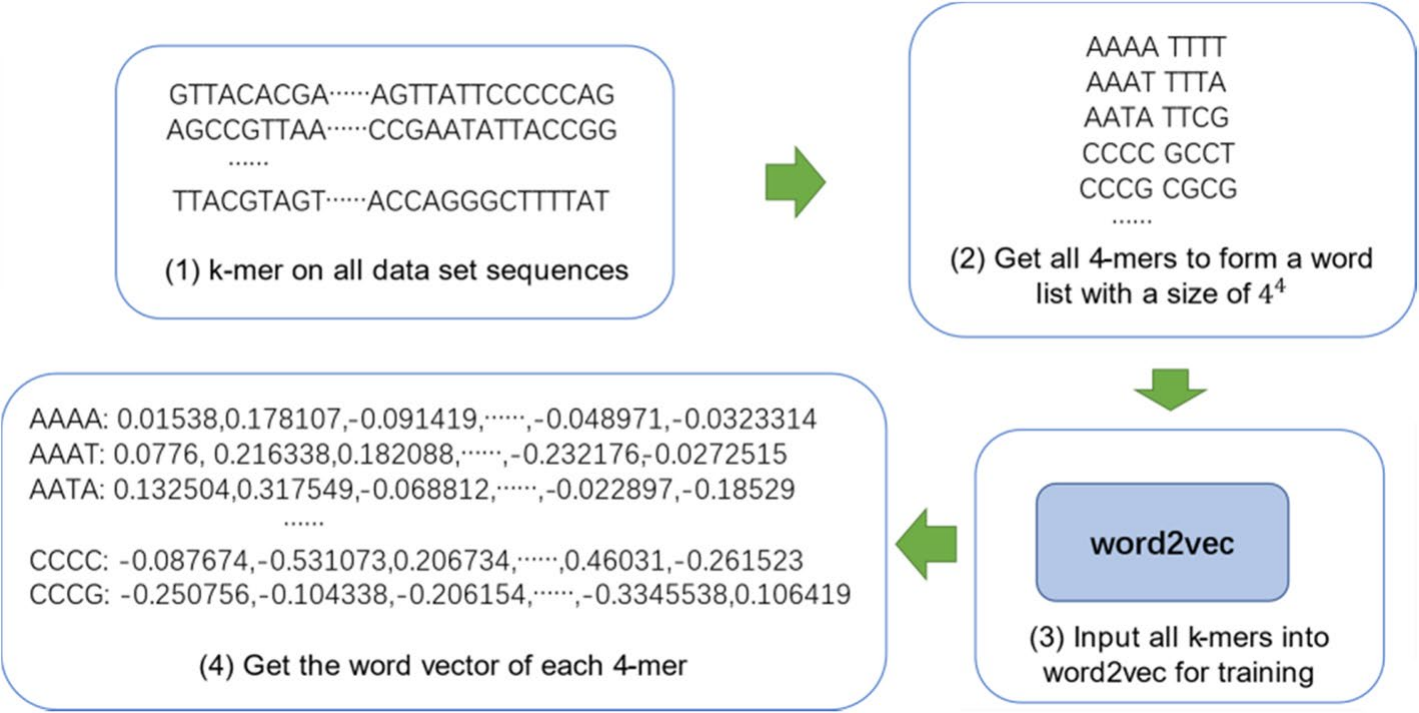
DNA sequence word vector representation flowchart

To apply word2vec technology to represent DNA sequences, it is necessary to segment the sequences into k-mers firstly [22]. It means that a DNA sequence is divided into substrings containing k bases [15], a sequence with length L is generally divided into L-k + 1 k-mers. We know that the number of all possible combinations of A,C,G,T for 4 digit is ${4}^{k}$, so the vocabulary size is ${4}^{k}$. All k-mers in a super large dataset are input into the model for training, then a word vector dictionary of ${4}^{k}$ k-mers can be obtained. According to the dictionary, each k-mer of a DNA sequence can be represented by a word vector, so that a length L DNA sequence can be converted into an embedding matrix. Taking 4-mer as an example, the process of word vector representation of DNA sequence is shown in Fig. 3.

### CNN model

Convolutional Neural Network (CNN) is a classic model in deep learning, which has shown extraordinary advantages in computer vision [30, 31]. It can also be applied in text classification tasks [25]. Convolutional layer is the core of CNN, and it performs convolution operations through filters to extract features from the input data. Meanwhile, the parameters in the convolutional layer are shared, which greatly reduces parameter scale. Pooling layer reduces the feature dimension by sampling the output, and it is often connected after convolutional layer. Pooling operation can not only simplify the network parameters and reduce the amount of calculation, but also further compress the features and key output features to prevent the model from overfitting. There are two common types: max pooling and average pooling.

**Fig. 4:**
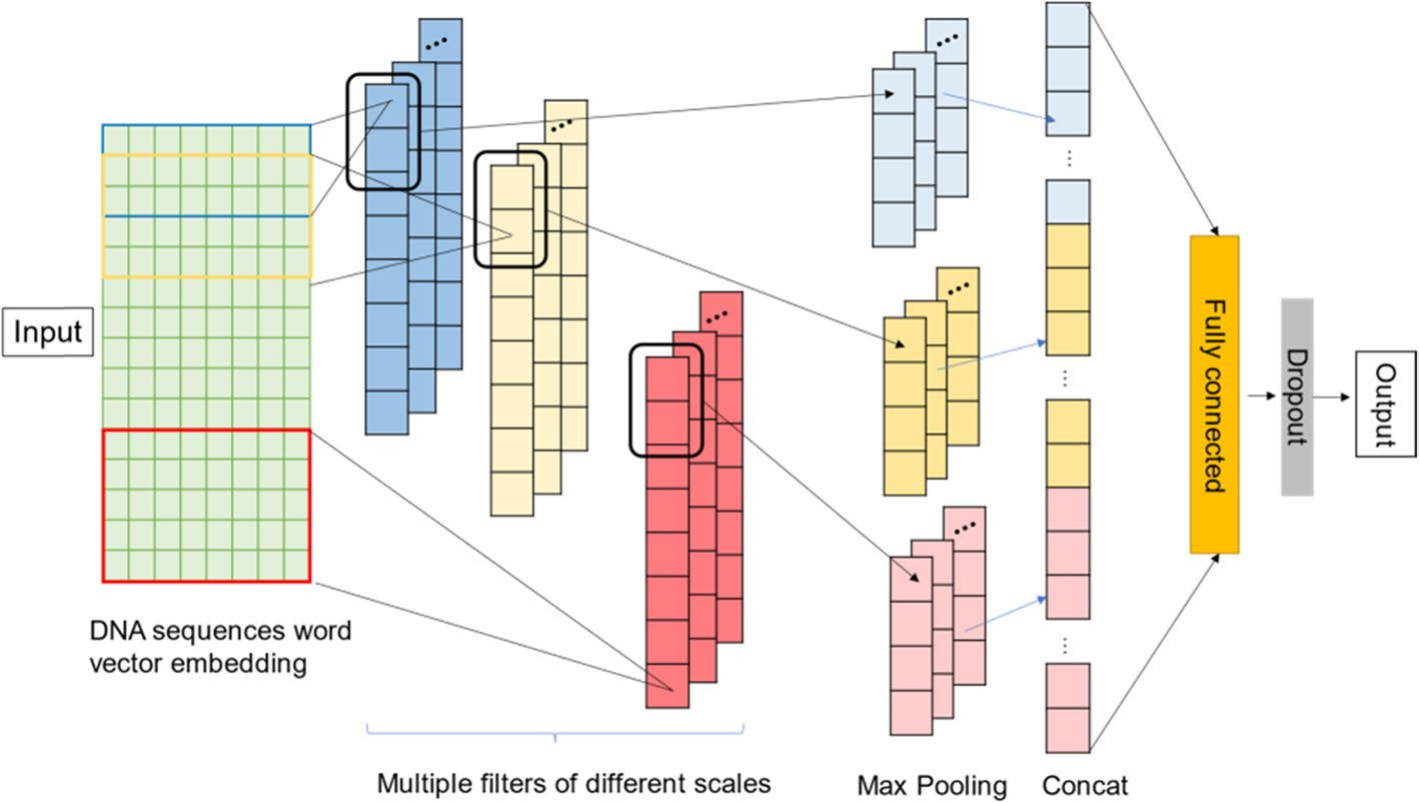
Nucleosome positioning model based on CNN and word vector

We establish nucleosome positioning prediction model based on the TextCNN, as shown in Fig. 4. Recently, DeepInsight [32] can perform non-image to image transformation, and DeepFeature [33] can also find features/genes other than non-image to image transformation which can be then used by CNN. More clearly and concisely, we use pre-trained word vectors of DNA sequences as inputs of the model, several different size of filters (3, 4, 5) for convolutional operation, and the number of filters is 64. Unlike TextCNN, the model changes global max pooling to max pooling with width and stride 2. This is more conducive for further extracting salient features and reducing the size of output features [34]. The fully connected layer contains 100 neurons, and the dropout ratio is 0.5 [35]. Batch size is 64, and the number of training iterations is 10 epochs, with a learning rate of 0.001. We use Adamax optimizer and cross-entropy loss function.

### BiGRU and BiLSTM model

The neurons of the hidden layer in recurrent neural network (RNN) are connected to each other so that the network is endowed with memory ability, which can mine the information hidden in the previous part of sequence. Therefore, RNN is mostly applied in sequence processing or generation tasks [36]. In particular, the bidirectional recurrent neural network (BiRNN) can also take the context into account, and integrate previous and future information, so generally it has a better efficiency. In this work, we try to construct the RNN model using two types of RNN units: LSTM and GRU [37].

**Fig. 5:**
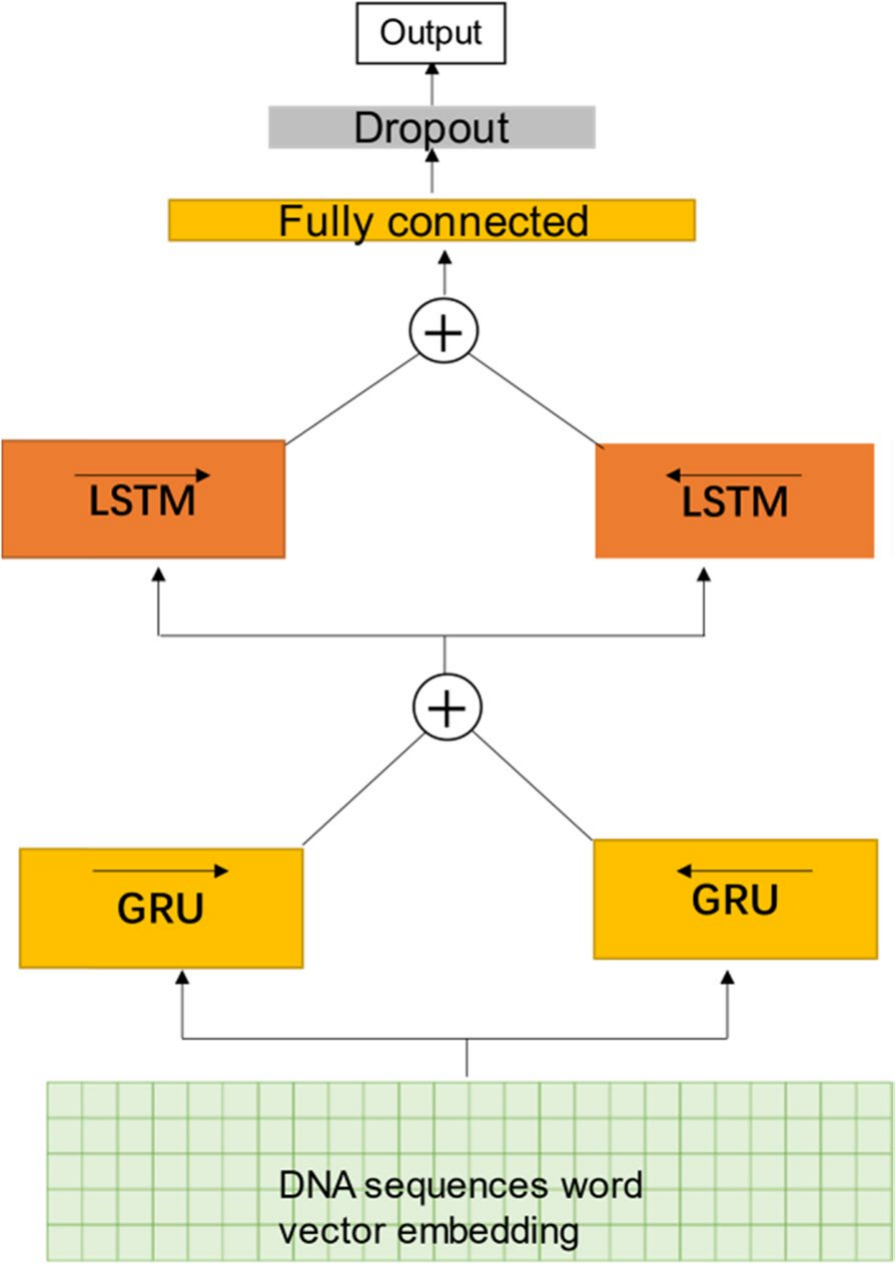
Nucleosome positioning model based on BiGRU + BiLSTM and word vector

LSTM unit is composed of three gates and a memory cell, which is responsible for the storage of information. The element value of each gate is between 0 and 1 to implement forgetting or strengthening [18]. The performance of GRU is almost equivalent to LSTM. While its parameter scale is much lower than LSTM, and it can also achieve long short-term memory function. GRU does not use the memory cell and three gates like LSTM but uses the update gate and the reset gate [38]. Considering that the sequence of bases in the DNA sequence contains hidden long-range correlation information, we constructed the model based on BiGRU and BiLSTM, as shown in Fig. 5.

The input layer of the model is followed by a bidirectional GRU layer. The output vector after bidirectional GRU is spliced and then input to a bidirectional LSTM layer, the information lost in the previous layer is further captured through LSTM network. The output features of bidirectional LSTM are connected together and input to a fully connected layer containing 100 neurons, and then a dropout layer (*p* = 0.5). Finally, a softmax fully connected layer is added for classification. The hidden size of GRU and LSTM are 100 and 200 respectively. Batch size is 64, and the number of training iterations is 15 epochs, with a learning rate of 0.001. We use Adamax optimizer and cross-entropy loss function here.

### Architecture of NP_CBiR

**Fig. 6:**
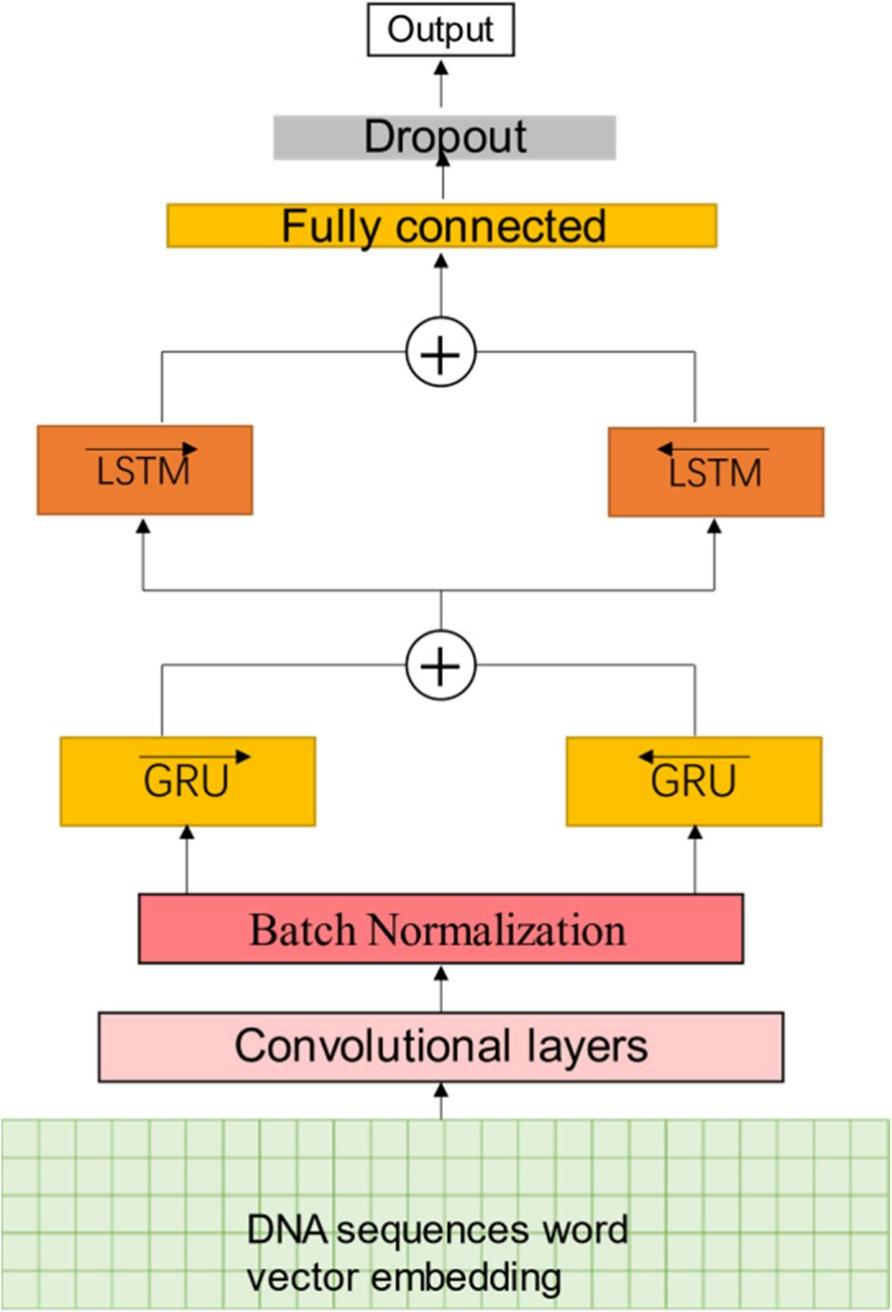
Nucleosome positioning model based on hybrid model and word vector

Some studies have shown that integrative models with multiple network structures have better capabilities of feature extraction [19, 20, 37]. Considering the model characteristics of CNN and RNN, we propose a hybrid model named NP_CBiR, as shown in Fig. 6.

NP_CBiR has been further modified on the basis of previous models. The specific content is as follows: In the convolutional layer, NP_CBiR only use one scale filter, the size is 5 with the number of 50. Although the sampling operation of pooling layer can reduce the feature dimension, it has the risk of destroying the global features. Since each segment in the DNA sequence is equally important, NP_CBiR uses batch normalization (BN) layer to replace pooling layer [39]. The normalization of the BN layer can effectively prevent the model from overfitting and improve the generalization ability. The network structure after BN layer is similar to Section D. The hidden sizes of GRU and LSTM are 50 and 100, respectively. The fully connected layer contains 100 neurons, and the dropout ratio is 0.5. Batch size is 64, and the number of training iterations is 15 epochs, with a learning rate of 0.0001. We also used Adamax optimizer and cross-entropy loss function.

## Data Availability

The datasets of this work can be downloaded from two published papers [12, 28]. The python source code used in this work are freely available at https://github.com/lliqi-echo/Nucleosome-positioning-based-on-DNA-sequence-word-vector-and-deep-learning.

## Abbreviations

ACC: Accuracy
AUC: Area under curve
BiRNN: Bidirectional recurrent neural network
BN: Batch normalization
CNN: Convolutional neural networks
GRU: Gated recurrent unit
LSTM: Long short-term memory
MCC: Mathew's correlation coefficient
RNN: Recurrent neural network
S_n_: Sensitivity
S_p_: Specificity
SVM: Support vector machine
